# Adsorbed Water Promotes Chemically Active Environments on the Surface of Sodium Chloride

**DOI:** 10.1021/acs.jpclett.3c00980

**Published:** 2023-06-29

**Authors:** Xiangrui Kong, Ivan Gladich, Nicolas Fauré, Erik S. Thomson, Jie Chen, Luca Artiglia, Markus Ammann, Thorsten Bartels-Rausch, Zamin A. Kanji, Jan B. C. Pettersson

**Affiliations:** † Department of Chemistry and Molecular Biology, Atmospheric Science, University of Gothenburg, 41296 Gothenburg, Sweden; ‡ European Centre for Living Technology (ECLT), Dorsoduro, Calle Crosera, 30124 Venice, Italy; § Qatar Environment and Energy Research Institute, Hamad Bin Khalifa University, Post Office Box 31110 Doha, Qatar; ∥ Department of Environmental Systems Science, ETH Zürich, 8092 Zürich, Switzerland; ⊥ Laboratory of Atmospheric Chemistry, Paul Scherrer Institute, 5232 Villigen PSI, Switzerland

## Abstract

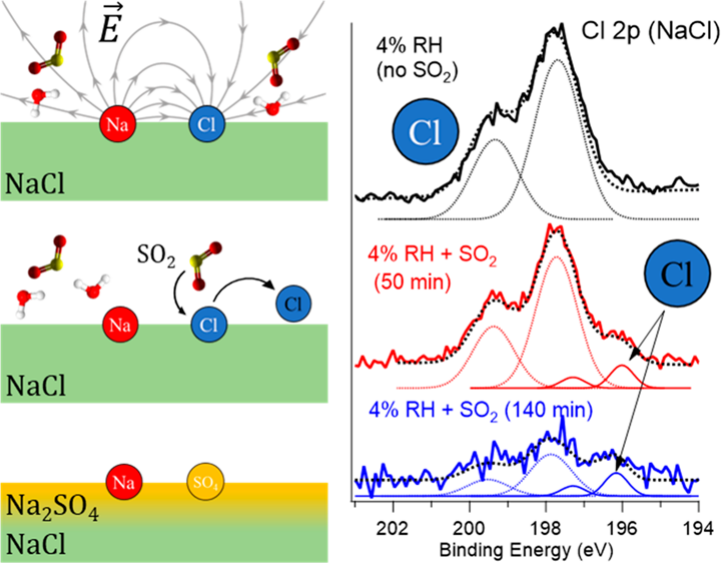

Gas–particle interfaces are chemically active environments. This study investigates the reactivity of SO_2_ on NaCl surfaces using advanced experimental and theoretical methods with a NH_4_Cl substrate also examined for cation effects. Results show that NaCl surfaces rapidly convert to Na_2_SO_4_ with a new chlorine component when exposed to SO_2_ under low humidity. In contrast, NH_4_Cl surfaces have limited SO_2_ uptake and do not change significantly. Depth profiles reveal transformed layers and elemental ratios at the crystal surfaces. The chlorine species detected originates from Cl^–^ expelled from the NaCl crystal structure, as determined by atomistic density functional theory calculations. Molecular dynamics simulations highlight the chemically active NaCl surface environment, driven by a strong interfacial electric field and the presence of sub-monolayer water coverage. These findings underscore the chemical activity of salt surfaces and the unexpected chemistry that arises from their interaction with interfacial water, even under very dry conditions.

Gas–particle interfaces are chemically active places,^1^ especially when reversibly adsorbed water is present in equilibrium with water vapor.^2^ For example, thermodynamic reactions that are normally disfavored have been found to spontaneously take place on salt surfaces that are being solvated by adsorbed water.^2^ More generally, (salt) surfaces have special properties and chemical reactivities, but our holistic understanding is incomplete. Chloride salts, particularly NaCl, are common compounds on Earth and other planets.^3−5^ From a physicochemical perspective, NaCl plays an active role in our climate system, notably in aerosol growth that leads to cloud formation, as a result of its high hygroscopicity.^6^ Furthermore, NaCl can bind with water molecules to hydrohalite (NaCl·2H_2_O),^7^ which serves as active ice nucleating particles (INPs).^8^ Nonetheless, chemically speaking, NaCl in its dry salt crystal form is generally considered to be unreactive, except for its surface, which can react with nitrogen oxides.^9^ Considering that the NaCl surface already exhibits surface-bound water at a quite low relative humidity (RH)^10^ and the potential that water may chemically activate the surface environment,^2^ it is relevant to understand the physicochemical nature of NaCl surfaces in the presence of adsorbed water.

In this study, the NaCl surface chemical activity is investigated in conjunction with SO_2_ gas uptake and the resulting transformations, which has a high relevance on the global scale given the considerable mixing of these two components. SO_2_ is a common and important gas from both natural and anthropogenic sources,^11,12^ which is particularly critical in aerosol science because SO_2_ gas can be efficiently converted to sulfate (SO_4_^2–^). Sulfate is an important contributor to particulate matter formation during haze periods.^13^ Traditionally, sulfate formation mechanisms primarily include gas phase oxidation of SO_2_ by OH radicals and aqueous oxidation of S(IV) by H_2_O_2_, O_3_, organic peroxides, NO_2_, and O_2_ catalyzed by transition metal ions in cloud/fogwater droplets.^14^ However, these mechanisms of oxidizing the precursor (SO_2_) to sulfate are insufficient to explain observed sulfate concentrations, especially in cloud-free, polluted areas where highly concentrated aerosol plays a role,^15^ which leads to large discrepancies in chemical transport models.^13,16^ This contributes to radiative forcing uncertainty and the role of SO_2_ therein, as shown in the latest Intergovernmental Panel on Climate Change (IPCC) report.^17^ When water is present on surfaces, surface catalysis mechanisms have been discovered to potentially have significant impacts on the sulfur chemistry of the atmosphere.^2,15,18,19^ Here, we investigate the heterogeneous pathway of SO_2_ uptake and conversion on the NaCl surfaces. For comparison, another chloride salt, NH_4_Cl, is used to examine the effects of a different cation and crystalline structure on the adsorption and chemistry. We combine surface-sensitive techniques [ambient pressure X-ray photoelectron spectroscopy (APXPS) and near-edge X-ray absorption fine structure (NEXAFS)] and first-principles molecular dynamics (FPMD) simulations to investigate the heterogeneous uptake and oxidation of SO_2_ gas on the surfaces of NaCl and NH_4_Cl.

In Figure 1, SO_2_ uptake on NaCl and the evolution of the system are shown, including the photoemission spectra of sulfur and chlorine species on NaCl and NH_4_Cl surfaces at various relative humidities (RHs) and after different exposure times (50 and 140 min) to 0.01 mbar SO_2_. On NaCl surfaces, the initial spectrum (spectrum α in Figure 1a, at a RH of 4%, without SO_2_) shows a neat doublet fitted with two component peaks at binding energies (BEs) of 199.4 and 197.7 eV, respectively. These correspond to the chloride ions (Cl 2p electrons) in the NaCl crystalline structure.^20^ After the NaCl surface is exposed to SO_2_ for 50 min (spectrum β in Figure 1a), the Cl 2p intensity decreases noticeably (spectrum β is scaled by a factor of 3). In addition, a new component emerges at a lower BE, which is fitted by a doublet with peaks at around 197.7 and 196.0 eV. At a longer exposure time (140 min), the chloride-related signal is mostly depleted (spectrum γ in Figure 1a, where a scaling factor of 6 is applied), but the new Cl doublet remains visible. This species is formed when SO_2_ is dosed but never significantly grows, even as the original chloride doublet decreases substantially within 140 min of exposure time. A possibility is that this new doublet may correspond to an intermediate steady-state species on the surface, having balanced pathways for both formation and a sink; i.e., this species might be continuously formed and subsequently depleted, such as by escaping to the gas phase. The identification of this new Cl doublet is discussed in conjunction with theoretical results later.

**Figure 1:**
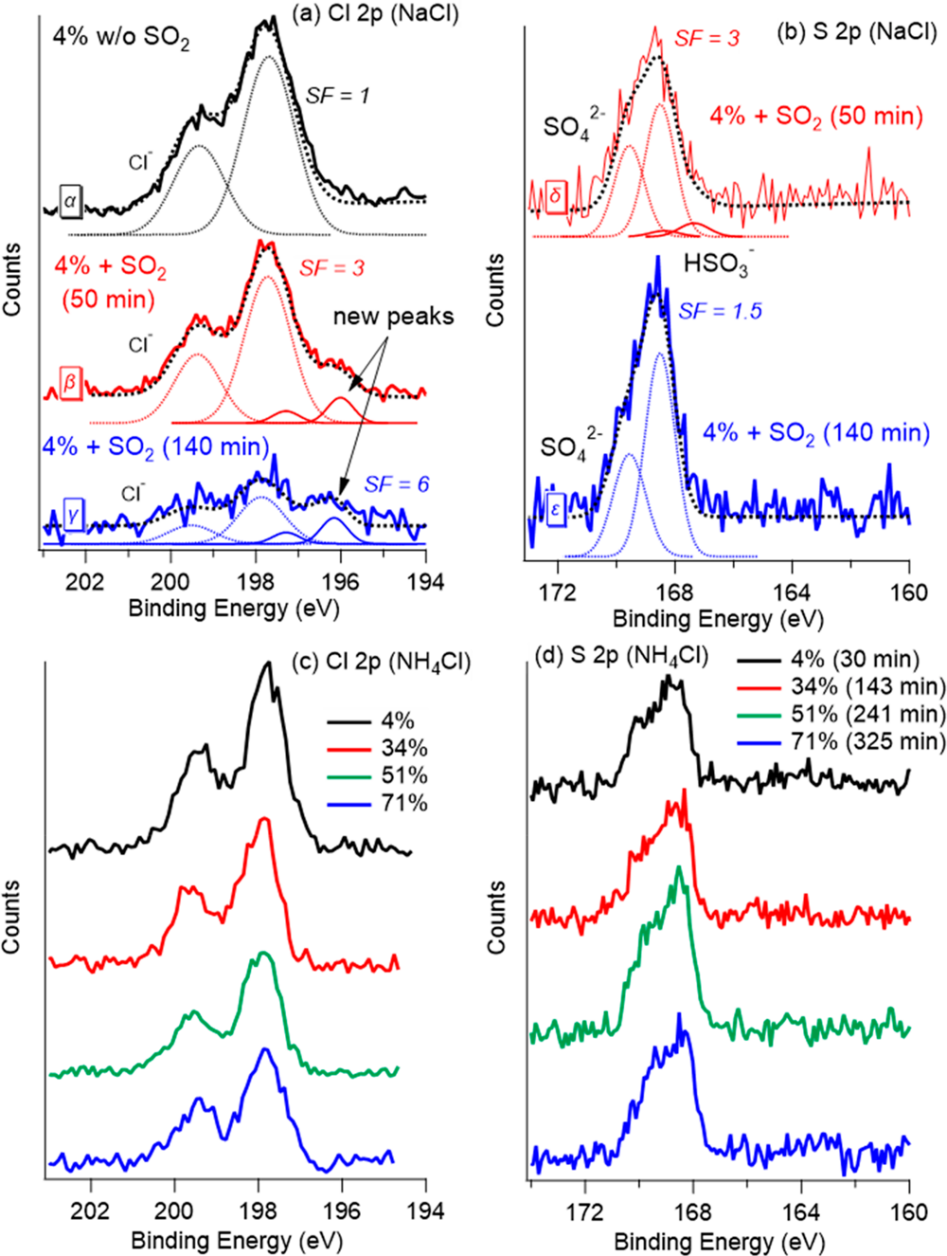
(a) Chloride 2p and (b) sulfur 2p XPS spectra measured at different exposure times (50 and 140 min) for 0.01 mbar of SO_2_ on NaCl at a RH of 4%. The photon energy was set to 570 eV for sulfur XPS and 600 eV for chlorine XPS, which results in the same electron kinetic energy for sulfur and chlorine and, consequently, the same mean escape depths. The spectra are normalized by scaling factors (SFs), which are marked beside the spectra. The fitted peaks are presented as dotted and solid lines. (c) Chlorine 2p and (d) sulfur 2p XPS spectra measured at different RHs with 0.01 mbar of SO_2_ exposure on NH_4_Cl. The exposure time is indicated in the legend. The binding energy was aligned using aliphatic carbon at 284.8 eV as a reference. During the measurements of these spectra, water and SO_2_ gases were present.

Sulfur spectra are shown in Figure 1b. The spectrum δ shows that, after 50 min, both sulfate and bisulfite (HSO_3_^2–^) doublets are observed on the surface (sulfate at 169.6 and 168.5 eV and bisulfite at 168.4 and 167.3 eV),^21−24^ showing the SO_2_ uptake and transformation on the surface. After 140 min, the sulfate doublet grew further (note the lower SF applied in spectrum ε). In addition, the soft X-ray radiation used in the experiments also plays a role and accelerates the displacement of chloride by sulfate, as discussed in Figure S1 of the Supporting Information.

In contrast, the chlorine depletion and sulfur accumulation are limited on the other investigated chloride salt, NH_4_Cl, even after long exposure times (>5 h) at various RHs (0–70%). Note that the different chemical reactivities of the two salts are not caused by the X-ray beam, because NH_4_Cl received a higher dose than NaCl. Panels c and d of Figure 1 show that the sulfate and chlorine signals are relatively stable over a large span of RHs, and no additional chlorine doublet emerges. The difference between the results for NaCl and NH_4_Cl salts shows that the NaCl surface is a more reactive environment for SO_2_ uptake, sulfate formation, and chlorine replacement.

The observation that chlorine is depleted as sulfate production increases supports a hypothesis that the NaCl surface is transforming to a surface composed of sodium and sulfate, e.g., Na_2_SO_4_. To verify this, sodium and oxygen K-edge NEXAFS spectra were acquired and are shown in Figure 2. For fresh NaCl that was never exposed to SO_2_, Figure 2a shows that its sodium NEXAFS has distinguishable peak features, as previously reported for crystalline NaCl.^25^ After SO_2_ exposure, the sodium NEXAFS spectra gradually change over time, toward a smoother line structure. For reference, the blue spectrum presents a pure Na_2_SO_4_ sample measured at a RH of 3%. After 5 h, the sodium NEXAFS spectrum has become very similar to the Na_2_SO_4_ reference spectrum, confirming that most sodium atoms are binding with sulfate instead of chloride on the surface. In addition, a “reverse” case is shown as the gray line, where the RH is reduced from 4 to 0% by stopping the water vapor supply. The RH drop does not induce any visible changes on the spectrum, suggesting that sulfate is stable at the surface. Moreover, sulfate remains stable under ultrahigh vacuum (UHV) conditions, because after being kept under UHV overnight, no sulfur depletion was observed.

**Figure 2:**
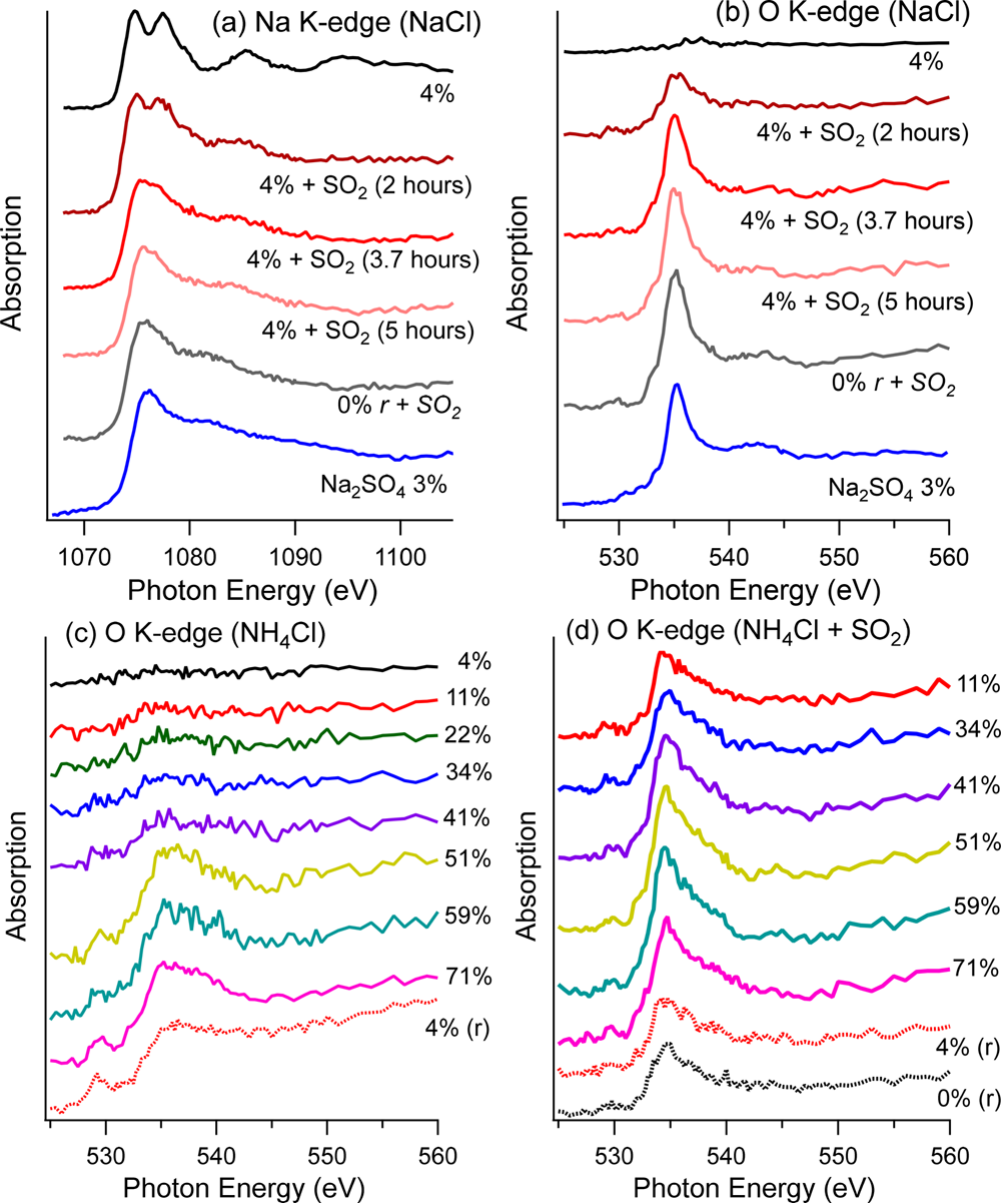
(a) Sodium K-edge and (b) oxygen K-edge NEXAFS measured at a RH of 4% with 0.01 mbar of SO_2_ exposure on NaCl. Oxygen K-edge NEXAFS was measured on NH_4_Cl at various RHs (c) without and (d) with 0.01 mbar of SO_2_ exposure. A detailed comparison of panels c and d can be found in Figure S2 of the Supporting Information.

The oxygen NEXAFS spectra (Figure 2b) show that there is almost no oxygen signal at a RH of 4% before the SO_2_ dosage. The trace amounts are likely attributable to adsorbed water that cannot be removed from the drop cast samples under these conditions. When SO_2_ is introduced, a sharp peak around 535 eV grows over time, which corresponds to oxygen in sulfate.^23,26^ Like the sodium NEXAFS spectra, after 5 h, the oxygen spectra show features similar to pure Na_2_SO_4_. Again, a RH reduction does not affect the sulfate in the oxygen spectrum. Noticeably, around 543 eV, a new structure does appear that, according to the energy, likely originates from water bonded to sulfate.^23^ This component is also maintained when the RH is reduced to 0%, indicating that strong bonds are formed.

To the contrary, the NH_4_Cl surface is relatively robust to SO_2_ exposure, independent of RH (panels c and d of Figure 2). In the SO_2_-free cases (Figure 2c), the oxygen NEXAFS shows that the surface is covered by adsorbed water when RH is increased above 40%, and the water remains when the measurement is taken as the RH returns to 4% (possibly a hysteresis effect). When SO_2_ is present (Figure 2d), oxygen NEXAFS shows a broad distribution already at a RH = 10%, which suggests the existence of a combination of adsorbed water and sulfate/bisulfite and highlights the synergetic effects of SO_2_ and water uptake. However, the sulfur abundance on the NH_4_Cl surface is stable over a wide RH range, as presented in Figure S3 of the Supporting Information. This implies that the NaCl surface is more active for SO_2_ uptake than NH_4_Cl at a RH of 4%.

Elemental ratios on the SO_2_-exposed NaCl surface are evaluated at two kinetic energies, and the results are shown in Figure 3a. All elemental ratios exhibit constant values between kinetic energies of 400 and 600 eV, which correspond to an electron inelastic mean free path (IMFP) of 1.2–1.6 nm in Na_2_SO_4_ and 1.5–2.0 nm for NaCl.^27^ The constant ratios indicate homogeneous distributions along the probed depth. Reference elemental ratios for pure materials (NaCl and Na_2_SO_4_) are marked as dotted lines, and quantitative chemometric analysis (see details in the Supporting Information) shows that the composition of layers is likely complicated, i.e., a combination of NaCl, Na_2_SO_4_, NaHSO_4_, and H_2_SO_4_.

**Figure 3:**
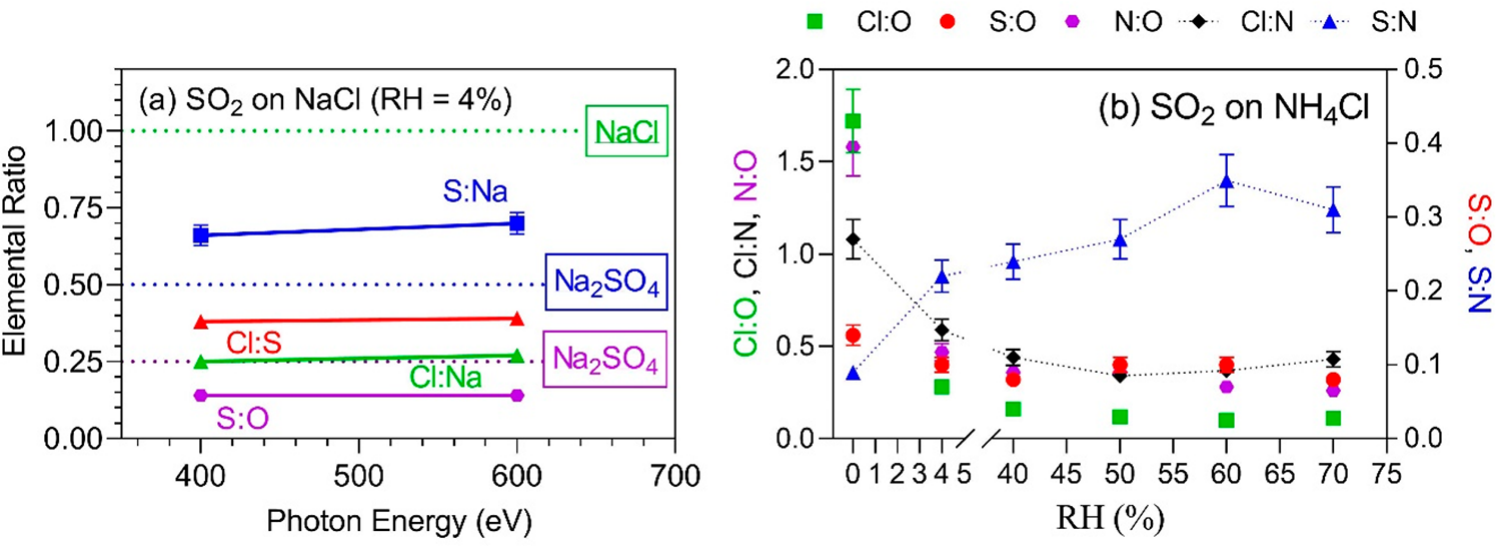
(a) Depth profile of elemental ratios of SO_2_-doped NaCl at a RH = 4%. The dotted lines show the elemental ratios of pure materials, as marked with boxes. The SO_2_ exposure time was 2–2.5 h during the measurements. (b) Depth profile of elemental ratios on a SO_2_-doped NH_4_Cl surface at various RHs. The measurement took about 8 h, and SO_2_ was present.

The elemental ratios for SO_2_ on NH_4_Cl are shown in Figure 3b as a function of RH. Some ratios, such as Cl/O, N/O, and Cl/N, decrease dramatically as RH increases and more water and SO_2_ are expected to co-condense on the surface. The decreasing Cl/N ratio indicates that Cl^–^ is selectively surface-depleted or NH_4_^+^ is relatively surface-enriched, because the only source of these elements is the salt itself. However, because gas phase SO_2_ can be taken up together with water vapor as RH rises, it is more likely that Cl^–^ is repelled from the gas–salt surface by competing anions, like SO_4_^2–^ or HSO_4_^–^. It is interesting that, on other hydrated salts (e.g., MgCl_2_·8H_2_O crystal), the observed behavior is the reverse, with monovalent ions drawn toward the water–salt interface to complete their solvation shells, while divalent ions are pushed toward the gas phase interface.^23^ The S/O ratio begins at 0.5 for RH = 0% as expected with only the SO_2_ present but quickly decreases with the presence or water vapor and then remains steady around 0.33, suggesting comparable uptake of the gas phase H_2_O and SO_2_. The only increasing trend shown in the figure is the S/N ratio, which rises together with RH as more SO_2_ is taken up at higher RHs.

To better understand the surface environment of NaCl and how relevant adsorbent species behave, atomistic simulations at density functional theory (DFT) levels were performed. The first task was to identify the chlorine species that appeared on the NaCl surface when exposed to SO_2_. Core electron binding energy (CEBE) calculations were performed for different chlorine species adsorbed on the surface of a NaCl ionic and cubic crystal slab with two open gas/crystal interfaces (see the Supporting Information for full details on the computational methodology). The considered compounds were Cl^•^ (Cl radical), Cl_2_, HCl, HSO_3_Cl, NCl_3_, NH_2_Cl, SCl_2_, and SO_2_Cl_2_. For the sake of efficient screening and as a result of the computational cost of the calculations, a single molecule was placed on the surface of the crystal at one time, as shown in Figure S3 of the Supporting Information. In addition, we tested the effect of the adsorption of hydronium (H_3_O^+^) and hydroxyl (OH^–^) ions on the CEBE of interfacial Cl atoms belonging to crystal NaCl (panels b and c of Figure S3 of the Supporting Information). Table S1 of the Supporting Information shows that, for all considered species, the Cl 2p lines lie at higher BE than those corresponding to Cl atoms belonging to the crystal substrate, indicating that none of them is the species responsible for the doublet at lower BE in Figure 1b. Moreover, this finding agrees with the general idea that CEBE increases when valence electrons are involved in bonds, because they reduce screening of nuclei attraction on the core electrons.^28^

Computational results help to clarify the replacement of chloride by sulfate at the surface of the NaCl crystal while also explaining the nature of the low BE doublet observed in Figure 1a. Figure S4 of the Supporting Information shows the energy profile obtained by the energy elastic band (see the Supporting Information for details) for the interfacial chloride–sulfate replacement. Interestingly, the BE of Cl atoms in the ionic lattice is higher than that of Cl^–^ ions expelled from the crystal (violet atom in the black and red insets of Figure S4 of the Supporting Information, respectively). This difference (Table S1 of the Supporting Information) is 2.2 eV, close to the experimental observation (about 1.6 eV). Generally, the BE of the core electrons increases when the valence electrons are involved in bonds,^28^ and the higher BE for Cl^–^ core electrons in the crystal may be due to a certain degree of electron sharing that is always present also in ionic bonds. This sharing reduces the screening of the valence electrons on the nuclei–core electron interaction, increasing the BE of core electrons in the crystal. On the other hand, when chloride is isolated, the screening of the valence electrons on the nucleus interaction is more effective, and thus, BE of core electrons is lower than in the mineral case. Figure S4 of the Supporting Information also shows a significant restructuring of the NaCl lattice and a very high energy barrier for the chloride–sulfate replacement in the absence of water. Interfacial dissolution of the NaCl crystal occurs in the presence of water,^29^ and the adsorption of few water molecules, even at low RH, are very likely to catalyze the interfacial chloride–sulfate substitution, lowering the high energy barrier for the chloride–sulfate replacement observed in Figure S4 of the Supporting Information in the absence of water.

To provide a molecular picture of the sub-monolayer solvation of the NaCl crystal, we performed 60 ps FPMD at 300 K, by placing 12 water molecules, 1 H_2_SO_4_, 1 H_2_SO_3_, 1 HCl, 1 Na_2_SO_4_, and 1 SO_2_ on the mineral surface (details in the Supporting Information). This system composition aims to resemble a possible scenario that may have been observed experimentally at low RH, with a water sublayer deposited on the surface of the crystals. Simulations show the ordering of water molecules in proximity to the water surface, a hectic proton dynamic, the formation of hydrogen bonds between water and solutes, hydroxyl and hydronium ions, and ion pairing (Figure S5 of the Supporting Information). Interestingly, the formation of hydroxyl and hydronium ions has also been reported at the interface of fully water-solvated NaCl crystals.^30^ Here, inspection of the trajectory surprisingly reveals spontaneous dehydration of sulfurous acid into SO_2_ (Figure 4a). Taken together, the simulations indicate that the partially solvated interfacial region of the crystalline surface is a very chemically active environment. A reason could be that, at the proximity of the mineral/water solution interface, solutes and solvent molecules are subject to an intense electric field, which may catalyze reactions.^31,32^Figure 4b shows the average component of the electric field along the direction (*z*) perpendicular to the crystal interface; the electric field profile shows two intense peaks, one localized at the mineral interface and one at the adsorbed water layer, while the electric field vanishes moving into the gas phase. The intensity of these peaks is 2.5 and 2 V/Å, respectively. The features shown in Figure 4b resemble those observed on other mineral–water interfaces,^31^ even if, in the present case, the high concentration and particular composition of the ionic species in the adsorbed water sub-monolayer play a substantial role in modulating the interfacial field. Nevertheless, it is crucial to note that, at the adsorbed water layer, the intensity of the electric field exceeds the value of 0.35 V/Å, at which water autoionizes.^33,34^ This very likely explains the hectic proton dynamics and reactivity of such an environment and also the spontaneous oxidation of SO_2_ on the NaCl surface without conventional oxidizing molecules observed experimentally. It is worth mentioning that, in the limited simulation time of computationally expensive FPMD, it was impossible to record the occurrence of Cl–sulfate replacement. Computational FPMD studies in the literature on the dissolution of a fully solvated NaCl crystal reported that more than 100 ps are needed to observe the initial disruption of the mineral lattice.^29^ In addition, the reactions were accelerated in the experiments by X-ray radiation, which is not considered in the simulation.

**Figure 4:**
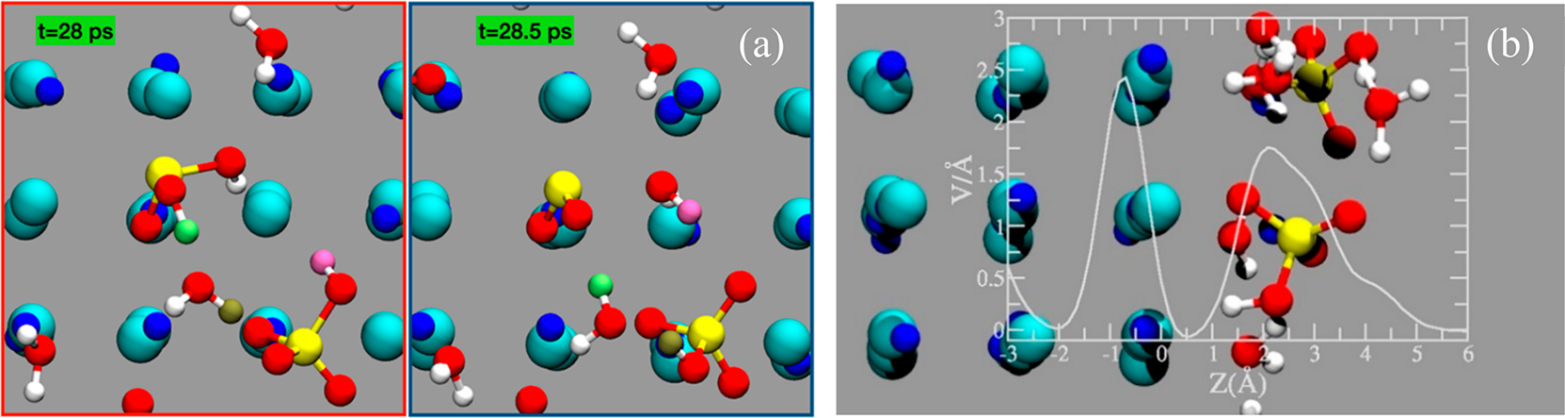
(a) Top view of a NaCl crystal showing the dehydration of H_2_SO_3_ into SO_2_. In green, brown, and pink, the H atoms are involved in proton transfer. (b) Average component of the electric field perpendicular to the interface, *E*_*z*_(*z*). A first peak is located in proximity to the interfacial NaCl crystal, and a second peak overlays the adsorbed water solution layer.

The results from this study show that salt surfaces can be reactive environments, providing the possibility for unconventional reactions under certain circumstances. The surface of NaCl is found to have a strong electric field, which is enough to autoionize water molecules. Such specific surface properties may explain the high chemical reactivities and SO_2_ uptake on chloride salts, especially on NaCl.

## Supporting Information Available

Materials and methods, chemometric analysis, experimental temperature and pressure, effects of soft X-ray on chloride–sulfate replacement, oxygen K-edge NEXAFS of NH_4_Cl, relative sulfur abundance, and computational methodology (PDF)
Transparent Peer Review report available (PDF)
