# Supplementary material for: Adsorbed Water Promotes Chemically Active Environments on the Surface of Sodium Chloride
Source: J Phys Chem Lett. 2023 Jun 29;14(26):6151–6. doi: 10.1021/acs.jpclett.3c00980 (PMC10331826; doi:10.1021/acs.jpclett.3c00980)
Supplement: Supplementary file 1 — jz3c00980_si_001.pdf [file jz3c00980_si_001.pdf]

Supporting Information

for

Adsorbed Water Promotes Chemically Active Environments on the Surface of Sodium Chloride

Xiangrui Kong[1*], Ivan Gladich[2,3*], Nicolas Fauré[1], Erik S. Thomson[1], Jie Chen[4], Luca Artiglia[5], Markus Ammann[5], Thorsten Bartels-Rausch[5], Zamin A. Kanji[4], and Jan B. C. Pettersson[1*]

[1]*Department of Chemistry and Molecular Biology, Atmospheric Science, University of Gothenburg, 41296 Gothenburg, Sweden*

[2]*European Centre for Living Technology (ECLT), Dorsoduro, Calle Crosera, 30124 Venice, Italy*

[3]*Qatar Environment and Energy Research Institute, Hamad Bin Khalifa University, P.O. Box 31110 Doha, Qatar*

[4]*Department of Environmental Systems Science, ETH Zürich, 8092 Zürich, Switzerland*

[5]*Laboratory of Atmospheric Chemistry, Paul Scherrer Institute, 5232 Villigen PSI, Switzerland*

Materials and Methods

The APXPS experiments were performed at the X07DB *In Situ* Spectroscopy beamline of the Swiss Light Source (SLS) at the Paul Scherrer Institute (PSI)[1]. The experimental cell was kept under high vacuum conditions (6×10^{-8} mbar base pressure) before and between measurements. The sample holder was loaded with a NaCl sample (ACS Reagent, ≥99.0%, Sigma-Aldrich) that was dissolved in ultrapure water (Fluka TraceSelect Ultra; Water ACS reagent) and deposited onto the holder via drop-casting. To accelerate water evaporation, the holder was warmed to 40°C. Once the sample was anhydrous, it was moved into the vacuum chamber. Water vapor was introduced from a temperature-controlled water reservoir via an 800 µm inner diameter stainless-steel capillary, and the water source (Fluka TraceSelect Ultra; Water ACS reagent) was degassed using 3 freeze-pump-thaw cycles. During the experiments, the relative humidity (RH) was adjusted by either changing the sample temperature while keeping the water vapor pressure constant or keeping the sample temperature constant while varying the water vapor pressure.

The X07DB beamline utilized a bending magnet as a photon source that generated photons ranging from 250 eV to 1500 eV, with a beam size of approximately 0.3 mm in diameter at the sample surface. The binding energy (BE) scale was aligned using aliphatic carbon at 284.8 eV as a reference. The acquired photoemission spectra were fitted using Gaussian functions after a linear background was subtracted. The results were used to normalize the photon flux, photoionization cross-sections, and analyzer transmission. Measured photoionization cross-sections were in good agreement with literature values. As a result, literature photoionization cross-sections and measured photon flux and analyzer transmission were utilized to normalize measured intensities. The photon dosage rate is between $1.1 \cdot 10^{14}$ to $5.6 \cdot 10^{14}$ photons·s^{-1}·cm^{-2}, depending on the photon energy.

Chemometric Analysis

In Figure 3a, the Cl:Na ratio is around 0.25, which is the fraction of NaCl to the total Na-containing compounds. Assuming the rest of Na is only associated with sulfate (Na_2SO_4), the S:Na ratio should be around 0.38, which is much lower than the measured value ≈ 0.70. This indicates that the sulfur is in excess with respect to Na, *i.e.*, there are other forms of sulfates that are associated with less or no Na, *e.g.*, $NaHSO_4$ and H_2SO_4. In two extreme cases, *i.e.*, Na_2SO_4 / $NaHSO_4$ mixture and Na_2SO_4 / H_2SO_4 mixture, the calculated molecular ratios are Na_2SO_4 / $NaHSO_4$ = 0.72, Na_2SO_4 / H_2SO_4 = 2.45, respectively. The actual composition of the system is likely complicated, so the real molecular ratio may be a combination of such extreme molecular ratios, Na_2SO_4, $NaHSO_4$, H_2SO_4, and NaCl.

Experimental Temperature and Pressures

Table S1 Actual water vapor pressure, surface temperature, equilibrium water vapor pressure and RH, which were used during the APXPS experiments.

Actual Water Pressure (mbar)	Surface Temperature (˚C)	Equilibrium Pressure (mbar)	RH
0	25	31.39	0%
1.35	25	31.39	4%
1.32	10	12.15	11%
1.3	0	6.04	22%
1.3	-6	3.86	34%
1.16	-10	2.83	41%
1.11	-13.2	2.19	51%
1.09	-15.3	1.84	59%
1.08	-17.6	1.52	71%

Effects of Soft X-ray on Chloride – Sulfate Replacement

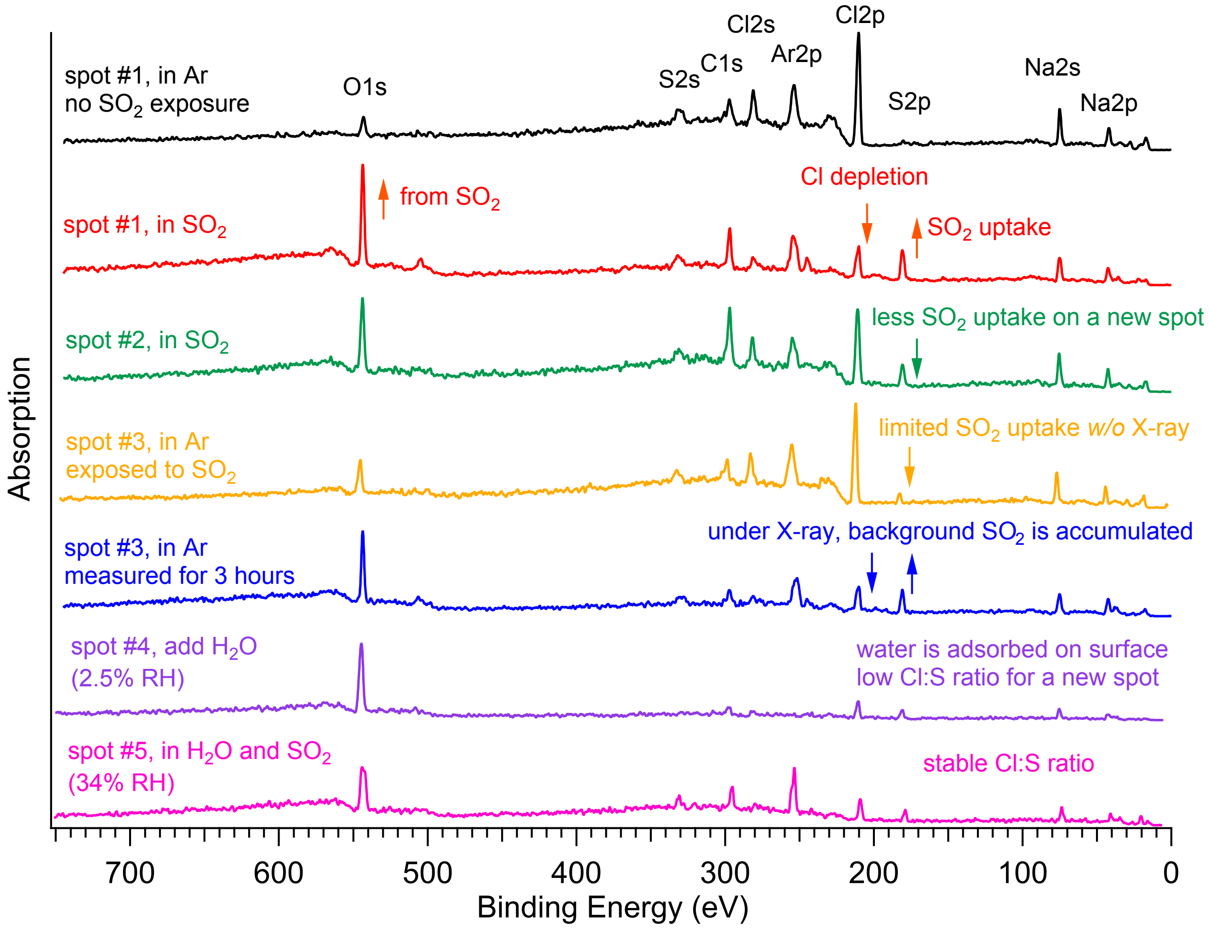

Figure S1 Survey spectra showing that the Cl depletion and SO_2 uptake on NaCl is facilitated by the X-ray. The sulfate formation is enhanced by the X-ray. Nevertheless, sulfate formation on NaCl has been observed, where the probed spot was never exposed to SO_2 and X-ray simultaneously (yellow line). SO_2 was first introduced but the spot was not measured, and then the measurement started after the SO_2 dosage was stopped and replaced by Ar gas.

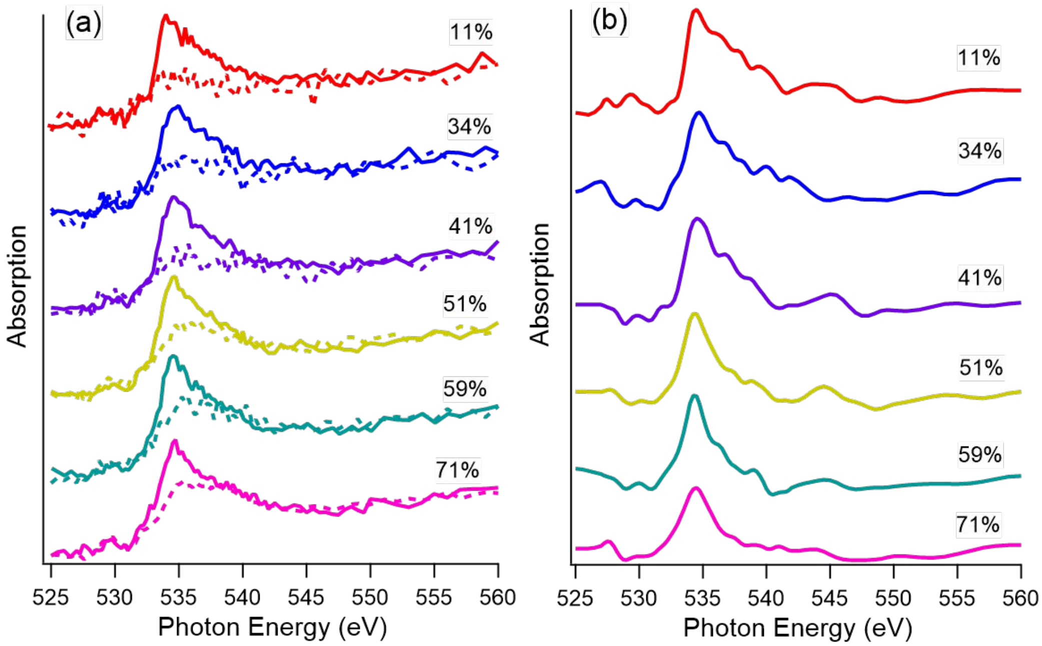

Figure S2 (a) Oxygen K-edge NEXAFS measured at various RH with and without 0.01 mbar SO_2 exposure on NH_4Cl, where the solid lines are for with SO_2 and the dashed lines are without SO_2. (b) Difference spectra between the solid and dashed lines showed in (a).

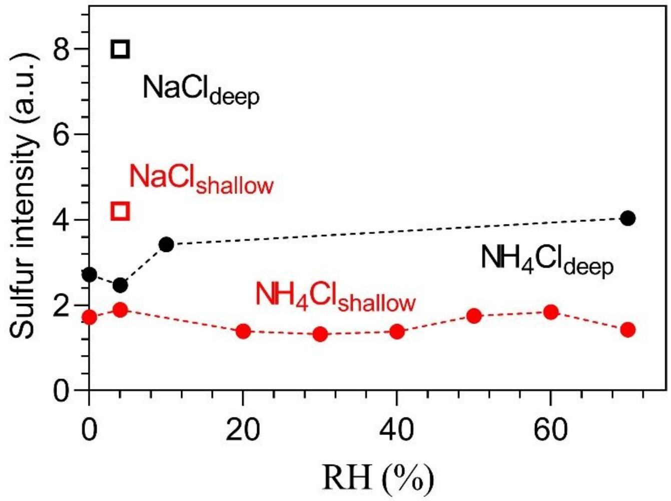

Figure S3 Relative sulfur abundance on NH_4Cl and NaCl surfaces as a function of RH. The figure shows the relative abundance of sulfur on the NH_4Cl surfaces at various RHs, where two probed depths are compared. The results show that the sulfur abundance is insensitive to RH, which remains relatively stable within the probed depth between RH 0% and 70%. The sulfur intensity is higher deeper in the surface because more atoms are probed and contribute to photoelectron yields. Two reference points of the NaCl case at RH = 4% (90 min SO_2 exposure) are included for comparison, which show a higher sulfur abundance on NaCl.

Computational Methodology

First principle molecular dynamics (FPMD) calculations based on density functional theory (DFT) were employed to determine the adsorption and dynamics of different compounds on the surface of the NaCl crystal and their spectroscopic signature. FPMD were employed using PBE[2-3] functional with Grimme dispersion correction (D3)[4] together with DZVP basis set. The basis set was truncated at a cutoff of 550 Ry. No XC-grid smoothing was used. The valence electrons were explicitly taken into account, while core electrons were treated by pseudopotentials[5]. For Na, the 9 valence electron basis representation was adopted. Similar set-up has been carefully tested (in term of cutoff and XC-grid smoothing options) in other simulations of solvated NaCl crystals.[6-7] Temperature was kept at 300 K using a Langevin thermostat[8] and a time constant of 300 fs. Time step was set to 0.5 fs. All FPMD simulations were performed using CP2K molecular dynamics package[9].

The initial NaCl (100) structure was constructed starting from the cubic crystal structure downloaded from Material Project Website (mp-22851).[10] The structure was then optimized at PBE-D3 level, resulting in a cell dimension of 17.47, 17.50 and 11.96 Å in the X,Y,Z dimension, respectively, with 72 Na and 72 Cl atoms. Afterward, the Z-dimension of the box was enlarged to 40 Å and further optimized. The final structure resulted in a NaCl slab with two mineral/vacuum interfaces and four NaCl layers perpendicular to the Z direction.

The experimental core electron binding energies (from XPS) has been compared with theoretically calculated density of states (DOS) at DFT level. By the virtue Koopmans'theorem[11] the relative positions among DOS peaks can be used to interpret experimental XPS spectra.[12-15] In particular, we explored different chlorine compounds (see Figure S1) adsorbed on the NaCl crystal, aiming to clarify the Cl2p peaks observed experimentally. The considered compounds were Cl• (Cl radical), Cl_2, HCl, HSO_3Cl, NCl_3, NH_2Cl, SCl_2 SO_2Cl_2. In addition, we also tested the effect of adsorbed hydronium (H_3O^+) and hydroxyl (OH^-) ions on the DOS line of interfacial Cl atoms belonging to the crystal substrate (Figure S1). After placing the compound on the crystal, the system was optimized at PBE-D3 level using a full cell optimization and, afterwards, DOSs were determined by all-electron calculations at PBE-D3 using the Gaussian Augmented Plane Wave (GAPW) scheme implemented in CP2K[16]. Pcseg-3 basis set,[17-18] which is a basis set explicitly designed for core ionization in combination with DFT, was adopted for all the species. The calculated DOS were smeared using a 0.5 eV Gaussian smearing: the 0.5 eV value was selected because it resembles the smearing generally observed experimentally and, moreover, is a conservative choice.[12, 15] Similar strategy for the DOS calculations has been adopted elsewhere.[12, 15]

Molecular dynamics (MD) simulations at *first-principles* level (FPMD), i.e., with forces driving the dynamics calculated "*on-the-fly*" by DFT,[19] were exploited to determine the solvation and dynamics of a water sublayer solution on one of the NaCl crystal surface. We placed 12 water

molecules with 1 H_2SO_4, 1 H_2SO_3, 1 HCl, 1 Na_2SO_4, and 1 SO_2 on the mineral surfaced (see Figure S3a), relaxing the system by geometry optimization. Afterwards, we exploited 60 ps using PBE[2-3] functional with Grimme dispersion correction (D3)[4].

The energy barrier required for the interfacial Cl-sulfate replacement was calculated using an energy path optimization by climbing image Nudged Elastic Band (CI-NEB), as implemented in CP2K.[9] The CI-NEB was performed using 9 replicas, without optimizing (the already optimized) band end points (*i.e.*, snapshot (a) and (b) in figure S2). The CI-NEB stopped when we recorded a maximum difference among the last 15 energy profiles that did not exceed the 0.5 kcal/mol.

The average z-component of the electric field in the system was determined by following the procedure outlined in ref.[20] Due to the symmetry of our system, with two crystal interfaces normal to the z-direction, we have chosen to look at the $E_z(x,y,z)$ component of the electric field. This component was average in the x, and y directions

$$E_{av,z}(z) = \frac{1}{A}\int dx \int dy \, E_z(x,y,z)$$

where A is the surface area of the simulation box. $E_{av,z}$ was calculated for 20 different configurations taken from the last 20 ps of our 60 ps-FPMD trajectory, dumping the trajectory every 1 ps. The average over the 20 snapshots, $E_{20\text{-}av,z}$, was finally filtered to eliminate oscillations in the bulk of the crystal by

$$< E_z > (z) = \frac{1}{b}\int_{z-b/2}^{z+b/2} dz' E_{20-av,z}(z')$$

where b was the inter-distance between ionic layer in the NaCl crystal (~3Å). Figure 4b reports $<E_z>(z)$

Table S1: Cl_{2p} CEBE line for different chlorine species on the top of the NaCl lattice. H_3O^+--Cl^- and OH^- - Cl^- refers to the line of the Cl atom at closer distance to the hydronium (hydroxyl) ion. Δ is the CEBE energy difference between Cl^- in the ionic lattice and the Cl of the adsorbed specie.

Cl_{2p}	CEBE(eV)	Δ
Cl^-	182.2	0
Cl*	183.2	1
Cl_2	184.5	2.3
H_3O^+--Cl^-	183.4	1.2
HCl	183.4	1.2
HSO_3Cl	184.5	2.3
NCl_3	184.6	2.4
NH_2Cl	184.5	2.3
OH^---Cl^-	182.9	0.7
SCl_2	185.2	3
SO_2Cl_2	185	2.8
Cl^- (out)		-2.2

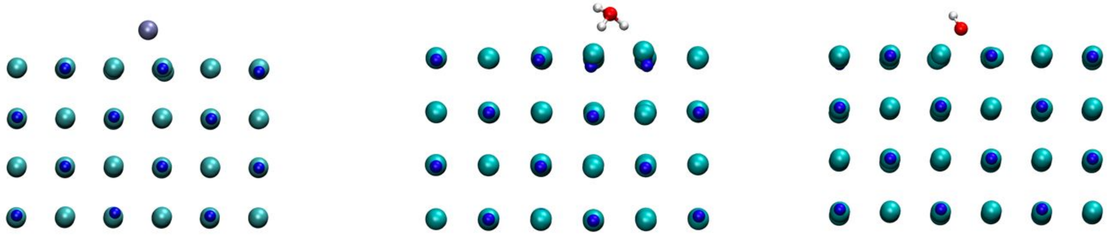

(a) (b) (c)

Figure S3: (Sample of) configurations used for the Cl_{2p} CEBE calculations of different chlorine species adsorbed on the surface of a NaCl crystal. Cl radical, H_3O^+ and OH^- adsorption on the mineral surface are shown in Panel (a), (b), and (c), respectively. Color code: S(yellow), O(red), H(white), Na (blue), Cl(cyan).

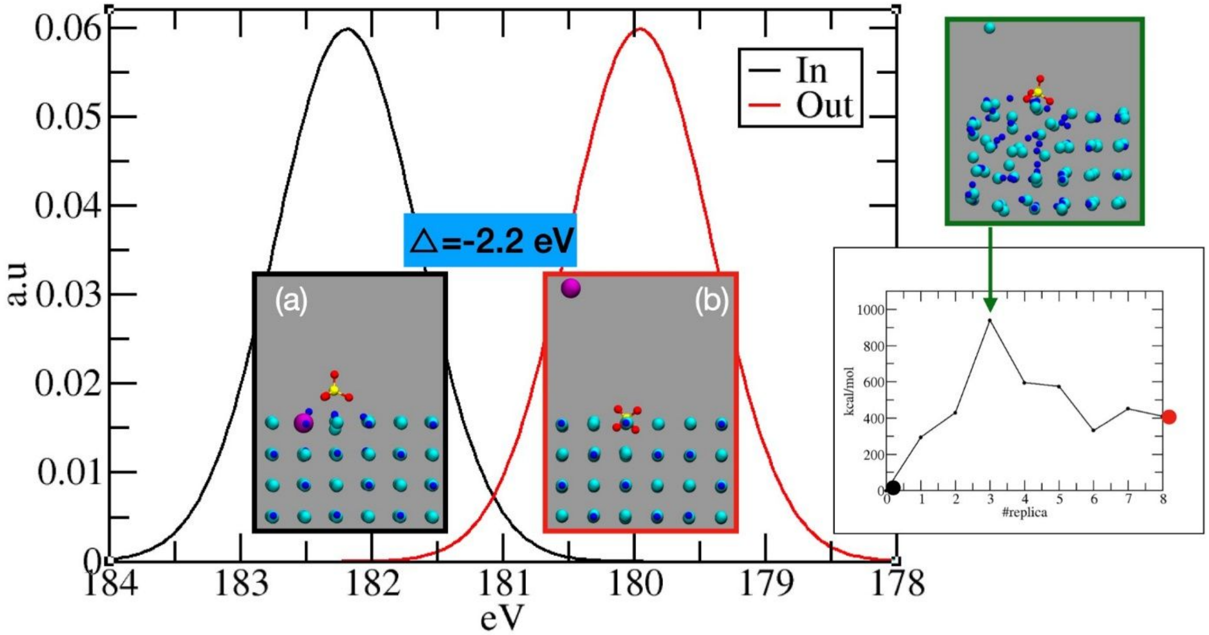

Figure S4: CEBE for a Cl atom inside the ionic crystal (snapshot-a) and outside the crystal structure (snapshot-b). The inset show the energy profile from snapshot (a) to snapshot (b) obtained by NEB.

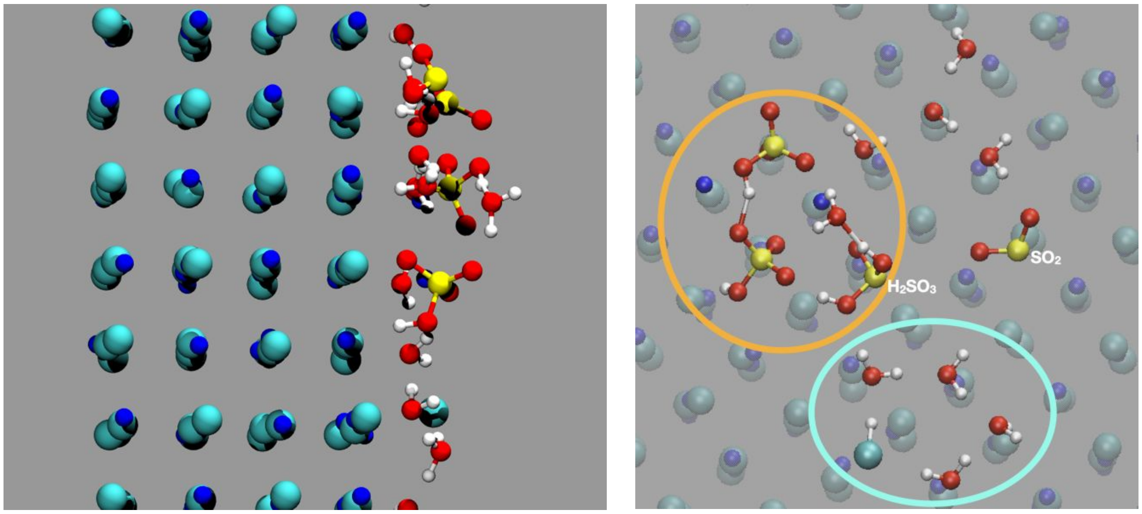

(a) (b)

Figure S5: Panel a): cross section of the system after 60 ps, along the Z direction perpendicular to the crystal interface. Panel (b): snapshot of the system showing proton transfer among Na_2SO_4, H_2SO_4, water and H_2SO_3 (orange circle) and water hydrogen bonding with HCl (cyan circle). Color code: S(yellow), O(red), H(white), Na (blue), Cl(cyan).

Reference:

1. Orlando, F.; Waldner, A.; Bartels-Rausch, T.; Birrer, M.; Kato, S.; Lee, M.-T.; Proff, C.; Huthwelker, T.; Kleibert, A.; van Bokhoven, J.; Ammann, M., The environmental photochemistry of oxide surfaces and the nature of frozen salt solutions: A new in situ XPS approach. *Top. Catal.* **2016**, *59* (5-7), 591-604.
2. Perdew, J. P.; Ruzsinszky, A.; Csonka, G. I.; Vydrov, O. A.; Scuseria, G. E.; Constantin, L. A.; Zhou, X.; Burke, K., Restoring the density-gradient expansion for exchange in solids and surfaces. *Phys. Rev. Lett.* **2008**, *100* (13), 136406.
3. Perdew, J. P.; Burke, K.; Ernzerhof, M., Generalized gradient approximation made simple. *Phys. Rev. Lett.* **1996**, *77* (18), 3865-3868.
4. Grimme, S.; Antony, J.; Ehrlich, S.; Krieg, H., A consistent and accurate ab initio parametrization of density functional dispersion correction (DFT-D) for the 94 elements H-Pu. *J. Chem. Phys.* **2010**, *132* (15), 154104.
5. Goedecker, S.; Teter, M.; Hutter, J., Separable dual-space Gaussian pseudopotentials. *Phys. Rev. B* **1996**, *54* (3), 1703-1710.
6. Holmberg, N.; Chen, J.-C.; Foster, A. S.; Laasonen, K., Dissolution of NaCl nanocrystals: an ab initio molecular dynamics study. *Phys. Chem. Chem. Phys.* **2014**, *16* (33), 17437-17446.
7. Kronberg, R.; Laasonen, K., Dynamics and surface propensity of H^+ and OH^- within rigid interfacial water: Implications for electrocatalysis. *J. Phys. Chem. Lett.* **2021**, *12* (41), 10128-10134.
8. Jones, A.; Leimkuhler, B., Adaptive stochastic methods for sampling driven molecular systems. *J. Chem. Phys.* **2011**, *135* (8), 084125.
9. Hutter, J.; Iannuzzi, M.; Schiffmann, F.; VandeVondele, J., cp2k: atomistic simulations of condensed matter systems. *WIREs Comput. Mol. Sci.* **2014**, *4* (1), 15-25.
10. Jain, A.; Ong, S. P.; Hautier, G.; Chen, W.; Richards, W. D.; Dacek, S.; Cholia, S.; Gunter, D.; Skinner, D.; Ceder, G.; Persson, K. A., Commentary: The materials project: A materials genome approach to accelerating materials innovation. *APL Mater.* **2013**, *1* (1), 011002.
11. Szabo, A.; Ostlund, N. S., Modern quantum chemistry: Introduction to advanced electronic structure theory. Dover Publications, Bethesda, Maruland, **1996**.
12. Pham, T. A.; Govoni, M.; Seidel, R.; Bradforth, S. E.; Schwegler, E.; Galli, G., Electronic structure of aqueous solutions: Bridging the gap between theory and experiments. *Sci. Adv.* **2017**, *3* (6), e1603210.
13. Gaiduk, A. P.; Govoni, M.; Seidel, R.; Skone, J. H.; Winter, B.; Galli, G., Photoelectron spectra of aqueous solutions from first principles. *J.A.C.S.* **2016**, *138* (22), 6912-6915.
14. Ping, Y.; Li, Y.; Gygi, F.; Galli, G., Tungsten oxide clathrates for water oxidation: A first principles study. *Chem. Mater.* **2012**, *24* (21), 4252-4260.
15. Kong, X.; Castarède, D.; Thomson Erik, S.; Boucly, A.; Artiglia, L.; Ammann, M.; Gladich, I.; Pettersson Jan, B. C., A surface-promoted redox reaction occurs spontaneously on solvating inorganic aerosol surfaces. *Science* **2021**, *374* (6568), 747-752.
16. VandeVondele, J.; Iannuzzi, M.; Hutter, J., Large scale condensed matter calculations using the Gaussian and augmented plane waves method. In *Computer simulations in condensed matter systems: From materials to chemical biology* Volume 1, Ferrario, M.; Ciccotti, G.; Binder, K., Eds. Springer Berlin Heidelberg: Berlin, Heidelberg, **2006**.
17. Ambroise, M. A.; Jensen, F., Probing basis set requirements for calculating core ionization and core excitation spectroscopy by the Δ self-consistent-field approach. *J. Chem. Theory Comput.* **2019**, *15* (1), 325-337.

18. Jensen, F., How large is the elephant in the density functional theory room? *J. Phys. Chem. A* **2017**, *121* (32), 6104-6107.
19. Marx, D.; Hutter, J., Ab initio molecular dynamics. Cambridge University Press, New York, **2009**.
20. Laporte, S.; Finocchi, F.; Paulatto, L.; Blanchard, M.; Balan, E.; Guyot, F.; Saitta, A. M., Strong electric fields at a prototypical oxide/water interface probed by ab initio molecular dynamics: MgO(001). *Phys. Chem. Chem. Phys.* **2015**, *17* (31), 20382-20390.
