# Supplementary material for: Adsorbed Water Promotes Chemically Active Environments on the Surface of Sodium Chloride
Source: J Phys Chem Lett. 2023 Jun 29;14(26):6151–6. doi: 10.1021/acs.jpclett.3c00980 (PMC10331826; doi:10.1021/acs.jpclett.3c00980)
Supplement: Supplementary file 2 — jz3c00980_si_003.pdf [file jz3c00980_si_003.pdf]

jz-2023-00980v.R1

Name: Peer Review Information for "Adsorbed Water Promotes Chemically Active Environments on the Surface of Sodium Chloride"

First Round of Reviewer Comments

Reviewer: 1

Comments to the Author

The paper is globally convincing; however, the following points should be better clarified (or corrected) by the authors:

1. Indicate the water pressure and substrate temperature, not only the RH

2. Although no ClO- (hypochlorite) forms, the authors should indicate what precautions are taken to avoid radiolysis phenomena. Precise the photon dose rate. maybe add in SI. This is a very useful information for people working in the field.

3. page 3 line 18, line 39. How many water molecules on the surface? Does a thin film form?

4. Precise how the binding energies are measured? with respect to contamination C 1s? Indicate in the SI the calibration procedure. This is important to convince the reader that the low BE Cl 2p doublet IS NOT Cl- and the high binding one ClO-....

5. Figure 1. The authors should precise whether the spectra are taken while the sample is exposed to the gas, or whether the hydration is performed in the dark (50-140 min) and then the NAP-XPS measurement is made.

6. Page 5 line 22. There spectrum delta. Correct the English

7. page 8. line 56. The discussion explaining the calculated BE between expelled Cl- and in site Cl- is not convincing. Especially the concept of screening. If the BE diminishes this means that the core-hole is better screened, not the contrary as stated. Where this expelled Cl- will go on the real surface? some kind of adatom? what about its possible hydration that is not taken into account.

8. p 9. line 49-50. Do the authors see spectral changes with time? If so, this should be reported in the SI

Reviewer: 2

Comments to the Author

Major Advance: The authors use surface sensitive spectroscopy (APXPS & NEXAFS) to determine the interaction between SO_2 and chloride salts under humid conditions. The authors find that NaCl is more

reactive than NH_4Cl. At low RH, NaCl reacts with SO_2 to form $(SO_4)^{2-}$, with a depletion of Cl^-. The amount of $(SO_4)^{2-}$ increases over time. In contrast, sulfate quickly accumulates on NH_4Cl under 4%RH, and the amount remains stable over time and over RH increases. Molecular dynamics simulations show water lowers the energy barrier to replace a Cl^- with a sulfate. the MD simulations and BE calculations help assign a low BE shoulder to solvated Cl^-.

Immediate impact: The high reactivity of NaCl with SO_2 under low RH conditions has several impacts. First, little fundamental chemistry is known about aerosol particles, which often contain salts and interact with SO_x pollutants. This study builds fundamental knowledge in atmospheric/environmental chemistry. Secondly, salts are thought to have little reactivity, especially at low RH. This study shows we must rethink this assumption. Finally, the paper's theoretical component shows the importance of the electric field at the solid surface on the surface chemistry. This finding is important as the understanding of the electronic structure of solid/liquid interfaces is evolving. I recommend that the paper be published following consideration of the few minor technical comments.

Technical Recommendations:

Fig 1 - The authors should show fits, otherwise the necessity of including a peak labeled as HSO_3^- is Fig. 1B is difficult to judge.

In Figure 3, please specify the SO_2 exposure time used for the data.

page 8, line 56, "stretching of the valence electrons" is a unfamiliar phrase to me, and it is not clear what the authors are intending. Please clarify.

The authors say Cl^- is solvated and removed from the surface on NaCl at 4%RH, and sulfate is replacing the Cl^-. What is the Cl^- counter ion?

Author's Response to Peer Review Comments:

Responses to reviewers' comments
Journal: The Journal of Physical Chemistry Letters
Manuscript ID: jz-2023-00980v
Title: "Adsorbed Water Promotes Chemically Active Environments on the Surface of Sodium Chloride"
Author(s): Kong, Xiangrui; Gladich, Ivan; Fauré, Nicolas; Thomson, Erik; Chen, Jie; Artiglia, Luca; Ammann, Markus; Bartels-Rausch, Thorsten; Kanji, Zamin A.; Pettersson, Jan

Editor:

As indicated in the enclosed comments, the reviewers' comments were generally positive, but certain improvements are suggested. I would be pleased to consider further for publication a revised manuscript that addresses the reviewers' concerns.
Reply: We appreciate the comments from the reviewers and the editor, which have been instructive and illuminated several areas for improvement and clarification within our manuscript. We have prepared a revised manuscript that thoroughly addresses the questions and comments that have been posed. Below we address and/or point to changes in the text for each reviewer comment in an itemized fashion. The reviewer comments are copied in plain text, while our responses and the actions we have taken are highlighted in blue.

Please also make the following non-scientific changes:

1. TOC Graphic: Please change/reformat the graphic and provide a TOC image per journal guidelines: The graphic should be in the form of a structure, graph, drawing, photograph, or scheme—or a combination. Non-scientific cartoon-like images or caricatures are discouraged.
   Action: A TOC image is now provided.

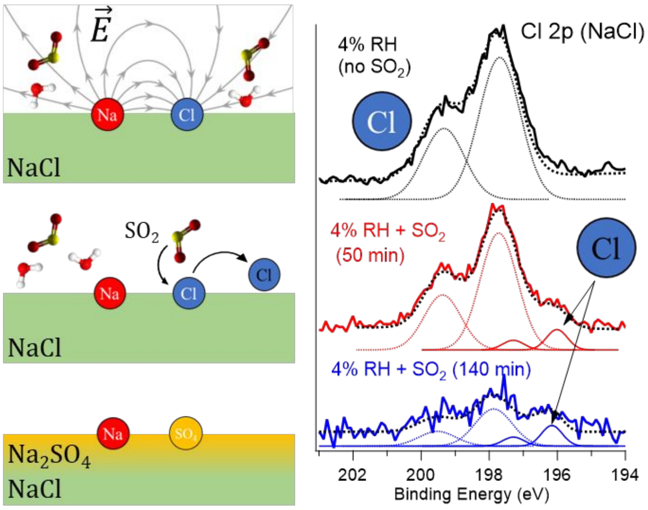

2. Abstract: Shorten the abstract to 150 words or fewer.
   Action: The abstract has been shortened.

3. Title and Author Lists: Title, author names, and affiliations must match in three places: (1) manuscript file, (2) supporting information, and (3) ACS Paragon Plus. Author names and list order do not match.
   Action: The author lists are now consistent.

4. References: In both the main file and the supporting information, fix the style of all references to use JPCL formatting (check all references carefully). ***JPC Letters reference formatting requires that journal references should contain: () around numbers, author names, article title (titles entirely in title case or entirely in lower case), abbreviated journal title (italicized), year (bolded), volume (italicized), and pages (first-last). Book references should contain author names, book title (in the same pattern), publisher, city, and year. Websites must include date of access.
   Action: The reference format has been checked and corrected.

5. Supporting Information: Please number SI pages in the following format: "S1, S2…"
   Action: Page numbers are now added.

6. Author Affiliations: Please include postal codes/country in the author affiliations in the publication file(s).
   Action: The missing postal code is added.

7. Supporting Information Statement: A brief, nonsentence description of the actual contents of each supporting information file is required. This description should be labeled Supporting Information and should appear before the Acknowledgement and Reference sections.
   Action: Supporting Information Statement has been added.

Reviewer: 1

The paper is globally convincing; however, the following points should be better clarified (or corrected) by the authors:

1. Indicate the water pressure and substrate temperature, not only the RH.
   Reply and Action: The water pressure and surface temperature have been added in a new table (Table S1).

2. Although no ClO^- (hypochlorite) forms, the authors should indicate what precautions are taken to avoid radiolysis phenomena. Precise the photon dose rate. maybe add in SI. This is a very useful information for people working in the field.
   Reply and Action: Although hypochlorite was not observed, the X-ray beam still accelerates the sulfate - chloride replacement. To understand this effect, we compared the two salts, NaCl and NH_4Cl, and the results show that the different chemical reactivities of the two salts are not because of the X-ray beam. Also, the effect of X-ray beamtime is illustrated in Figure S1, as stated in the text "...the soft

X-ray radiation used in the experiments also plays a role and accelerates the displacement of chloride by sulfate, as discussed in Figure S1 in Supporting Information.”. To emphasize the difference of the two salts, we now added the following text: “Note that the different chemical reactivities of the two salts are not caused by the X-ray, as the NH_4Cl received even longer exposure time than NaCl.”. The photon dose rate was between $1.1\cdot10^{14}$ to $5.6\cdot10^{14}$ photons$\cdot s^{-1}\cdot cm^{-2}$, depending on the photon energy. This information is now added in Supporting Information.

3. page 3 line 18, line 39. How many water molecules on the surface? Does a thin film form?
   Reply and Action: Under the conditions used in the cited reference studies, the salt surface was completely covered by water molecules, and the thickness of the coating is most likely of a microscopic/molecular character. However, from the cited studies it is not clear what water coverage is required to trigger an active surface environment.

   However, our computer simulations suggest that one sublayer of water is sufficient to activate chemistry, such as the spontaneous dehydration of sulfurous acid into SO_2. We have added the following sentence in the main manuscript: *“This system composition aims to resemble a possible scenario that may have been observed experimentally at low RH, with a water sublayer deposited on the surface of the crystals.”*

4. Precise how the binding energies are measured? with respect to contamination C 1s? Indicate in the SI the calibration procedure. This is important to convince the reader that the low BE Cl 2p doublet IS NOT Cl^- and the high binding one ClO-....
   Reply and Action: The binding energies were aligned using aliphatic carbon as a reference (C1s at 284.8 eV). This is added in the caption of Figure 1, to clarify the binding energy has been carefully aligned.

5. Figure 1. The authors should precise whether the spectra are taken while the sample is exposed to the gas, or whether the hydration is performed in the dark (50-140 min) and then the NAP-XPS measurement is made.
   Reply and Action: During the measurements of these spectra, the water and SO_2 gases were present. This information has been now added into the caption of Figure 1.

6. Page 5 line 22. There spectrum delta. Correct the English.
   Reply and Action: Corrected.

7. page 8. line 56. The discussion explaining the calculated BE between expelled Cl^- and in site Cl^- is not convincing. Especially the concept of screening. If the BE diminishes this means that the core-hole is better screened, not the contrary as stated. Where this expelled Cl^- will go on the real surface? some kind of adatom? what

about its possible hydration that is not taken into account.

Reply: We thank the reviewer for this comment, and we agree that the source of misunderstanding lies in the concept of screening.

Generally, BE of the core electrons increases when the valence electrons are involved in bonds because of the screening of the valence electrons, which are slightly more distant from the nucleus than the core electrons, and the core electrons/nuclei interaction is reduced. Even in an ionic crystal involving elements with distinctly different electronegativities, such as NaCl, in which valence electrons are transferred to complete the Lewis octet of each element, there is always some degree of electron sharing. Thus, BE of the core electrons in the mineral is higher than in isolated Cl^- because the screening of the valence electrons on the nuclei/core electrons is less effective in the crystal.

We change the text in the main manuscript as follows:
"*Generally, BE of the core electrons increases when the valence electrons are involved in bonds,*[28] *and the higher BE for* Cl^- *core electrons in the crystal may be due to a certain degree of electron sharing that is always present also in ionic bonds. This sharing reduces the screening of the valence electrons on the nuclei-core electrons interaction, increasing the BE of core electrons in the crystal. On the other hand, when chloride is isolated, the screening of the valence electrons on the nucleus interaction is more effective and, thus, BE of core electrons is lower than in the mineral case.*"

Regarding the second part of the comment about the location of expelled chloride: Our FPMD shows, indeed, that Cl^- remains adsorbed on the surface and hydrated by few water molecules (Figure S5b), within the (unavoidable limited) time scale of our simulations (60 ps). For reasons of computational time and methodology (e.g., Nudged Elastic Bands do not work well with too many degrees of freedom) Figure S4 shows the energy barrier for the chloride - sulfate replacement in absence of water. As stated in the main manuscript, the adsorption of a few water molecules, even at low RH, are very likely to catalyze the interfacial chloride - sulfate substitution.

8. p 9. line 49-50. Do the authors see spectral changes with time? If so, this should be reported in the SI.
   Reply and Action: The X-ray beam accelerates the transformation of the NaCl surface but does not significantly affect the NH_4Cl surface. Figure S1 illustrates this in the *Supporting Information*.

Reviewer: 2

1. Major Advance: The authors use surface sensitive spectroscopy (APXPS & NEXAFS) to determine the interaction between SO_2 and chloride salts under humid

conditions. The authors find that NaCl is more reactive than NH_4Cl. At low RH, NaCl reacts with SO_2 to form $(SO_4)^{2-}$, with a depletion of Cl^-. The amount of $(SO_4)^{2-}$ increases over time. In contrast, sulfate quickly accumulates on NH_4Cl under 4% RH, and the amount remains stable over time and over RH increases. Molecular dynamics simulations show water lowers the energy barrier to replace a Cl^- with a sulfate. the MD simulations and BE calculations help assign a low BE shoulder to solvated Cl^-.

2. Immediate impact: The high reactivity of NaCl with SO_2 under low RH conditions has several impacts. First, little fundamental chemistry is known about aerosol particles, which often contain salts and interact with SO_x pollutants. This study builds fundamental knowledge in atmospheric/environmental chemistry. Secondly, salts are thought to have little reactivity, especially at low RH. This study shows we must rethink this assumption. Finally, the paper's theoretical component shows the importance of the electric field at the solid surface on the surface chemistry. This finding is important as the understanding of the electronic structure of solid/liquid interfaces is evolving. I recommend that the paper be published following consideration of the few minor technical comments.

Technical Recommendations:

3. Fig 1 - The authors should show fits, otherwise the necessity of including a peak labeled as HSO_3^- is Fig. 1B is difficult to judge.
   Reply and Action: The overall fits are now added to Figure 1.

4. In Figure 3, please specify the SO_2 exposure time used for the data.
   Reply and Action: The SO_2 exposure time has been added in the caption of Figure 3.

5. page 8, line 56, "stretching of the valence electrons" is a unfamiliar phrase to me, and it is not clear what the authors are intending. Please clarify.
   Reply and Action: Please see reply #7 of reviewer #1.

6. The authors say Cl^- is solvated and removed from the surface on NaCl at 4%RH, and sulfate is replacing the Cl^-. What is the Cl^- counter ion?
   Reply and Action: The counter ion should be H^+, which neutralizes SO_4^{2-}/SO_3^{2-} before the sulfur – chlorine replacement. The final state of released Cl is likely be HCl, though it is not detected by XPS because it is in the gas phase.
